# A new criterion for assessing Ilizarov treatment outcomes in nonunion of the tibia

**DOI:** 10.1007/s00402-020-03571-8

**Published:** 2020-08-10

**Authors:** Łukasz Szelerski, Andżelika Pajchert Kozłowska, Sławomir Żarek, Radosław Górski, Karol Mochocki, Maciej Dejnek, Wiktor Urbański, Paweł Reichert, Piotr Morasiewicz

**Affiliations:** 1 Department of Orthopedics and Musculoskeletal Traumatology, Medical University of Warsaw, Lindeya 4, 02-005 Warsaw, Poland; 2 Department and Clinic of Orthopaedic and Traumatologic Surgery, Wrocław Medical University, ul. Borowska 213, 50-556 Wrocław, Poland; 3 Division of Sport Medicine, Department of Physiotherapy, Faculty of Health Sciences, Wroclaw Medical University, Bartla 5, 51-618 Wrocław, Poland

**Keywords:** Maintained union, Union rates, Nonunion, Tibia, Ilizarov method

## Abstract

**Introduction:**

The purpose of this study was to assess a population of patients with nonunion of the tibia treated with the Ilizarov method in terms of achieved union rates and maintained union rates, determination of re-fracture factors, with a subsequent comparison of our findings with those reported in the available literature.

**Materials and methods:**

This study was a retrospective assessment of 102 patients with nonunion of the tibia treated with the Ilizarov method in the period 2008–2015. The assessed parameters were bone union achieved during treatment, duration of stabilization with an Ilizarov external fixator, and maintained bone union at the last follow-up visit.

**Results:**

The mean age at the start of treatment was 46.7 years (11–84 years). The mean follow-up period was 7 years (2–12 years). Bone union was achieved in all patients. The mean duration of Ilizarov stabilization in the study group was 7.9 months (2.8–20.7 months). The rate of union maintained at the last follow-up visit was 95.1%.

**Conclusions:**

All patients in our study achieved bone union, which constitutes a better outcome than those reported on average in the literature (73.7–100%). The mean length of time which the Ilizarov external fixator was in place in our patients was 8.3 months, which is consistent with the data from literature. Infection, atrophic nonunion, nonunion in 1/3 distal of tibia, and close surgery technique are risk factors of re-fracture. None of the analyzed studies assessed the proportion of patients with maintained bone union. In our study, maintained bone union was observed in 95.1% of patients at the follow-up visit at least 2 years after treatment, which indicates excellent long-term treatment outcomes in nonunion of the tibia treated with the Ilizarov method.

## Introduction

Due to the anatomical structure and relatively poor perfusion in the distal third of the leg, fractures in this part of the body—in comparison with other locations—relatively commonly result in disturbed healing and nonunion [1–6]. In tibial fractures, nonunion rates range from 2.5 to 11% of cases [5, 6]. Ilizarov external fixators are an established technique for treating bone-healing disturbances [1–27]. The treatment aims to achieve bone union and painless, efficient gait, while focusing not only on the immediate outcomes. In fact, one equally important indicator of successful treatment is maintained bone union.

The literature on the subject comprises a number of papers on treating nonunion of the tibia with an Ilizarov external fixator [1–27]. The investigators focused on presenting various surgical techniques [2, 3, 5–11, 13, 16–19, 23, 26] and assessing the supportive role of various medications, means, and techniques in achieving bone union [1, 4]. These studies were predominantly concerned with assessing short-term treatment results [1–27]. For instance, the available literature on assessing Ilizarov method treatment outcomes in patients with nonunion of the tibia used such parameters as the rate of union, which is a short-term outcome [1–27]. Some patients who achieve union later develop re-fracture at the pseudarthrosis union site [1, 19], with the re-fracture rates as high as 31.6% [19]. These patients require retreatment, which translates to poor long-term outcomes.

Some authors who reported re-fracture rates considered them only as a complication, with no significant impact on treatment outcomes [1, 19, 25]. There are no studies on the long-term treatment outcomes (in terms of maintaining bone union over many years of follow-up) in nonunion of the tibia treated with the Ilizarov method. Thus, we would like to introduce a new criterion in assessing long-term outcomes, namely, maintained bone union. We believe the outcomes of treating nonunion of the tibia with the Ilizarov method which should be assessed comprehensively, both in terms of short-term (union rates—achieving union after treatment) and long-term parameters (maintained union rates).

The purpose of this study was to assess a population of patients with nonunion of the tibia treated with the Ilizarov method in terms of achieved union rates and maintained union rates, determination of re-fracture factors, with a subsequent comparison of our findings with those reported in the available literature.

## Materials and methods

**Fig. 1:**
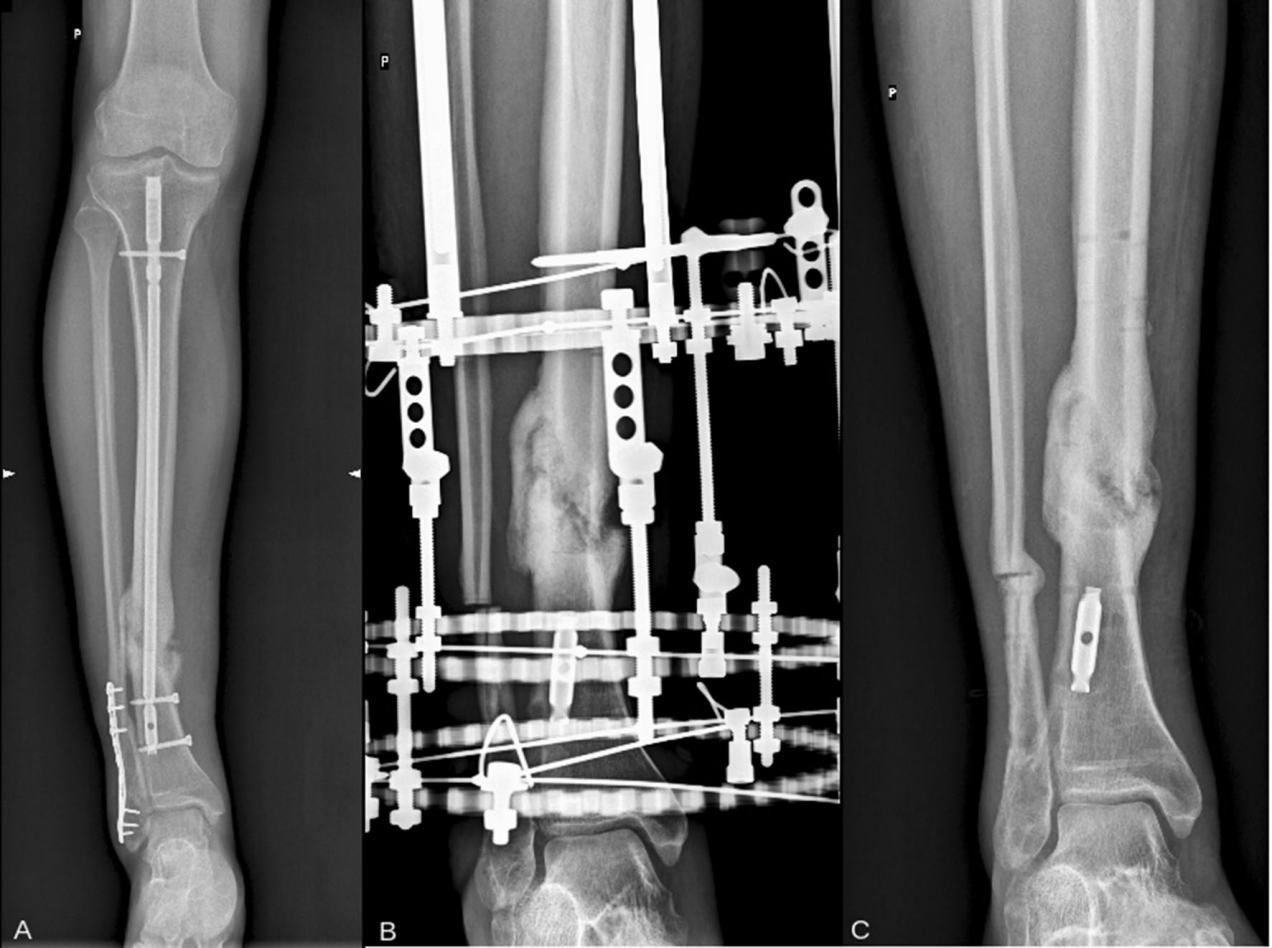
Patient with tibia nonunion. **a** X-ray from the pre-treatment period, **b** X-ray with Ilizarov apparatus after union, and **c** X-ray from the last follow-up visit after 6 years form apparatus removal, confirming maintained union

**Fig. 2:**
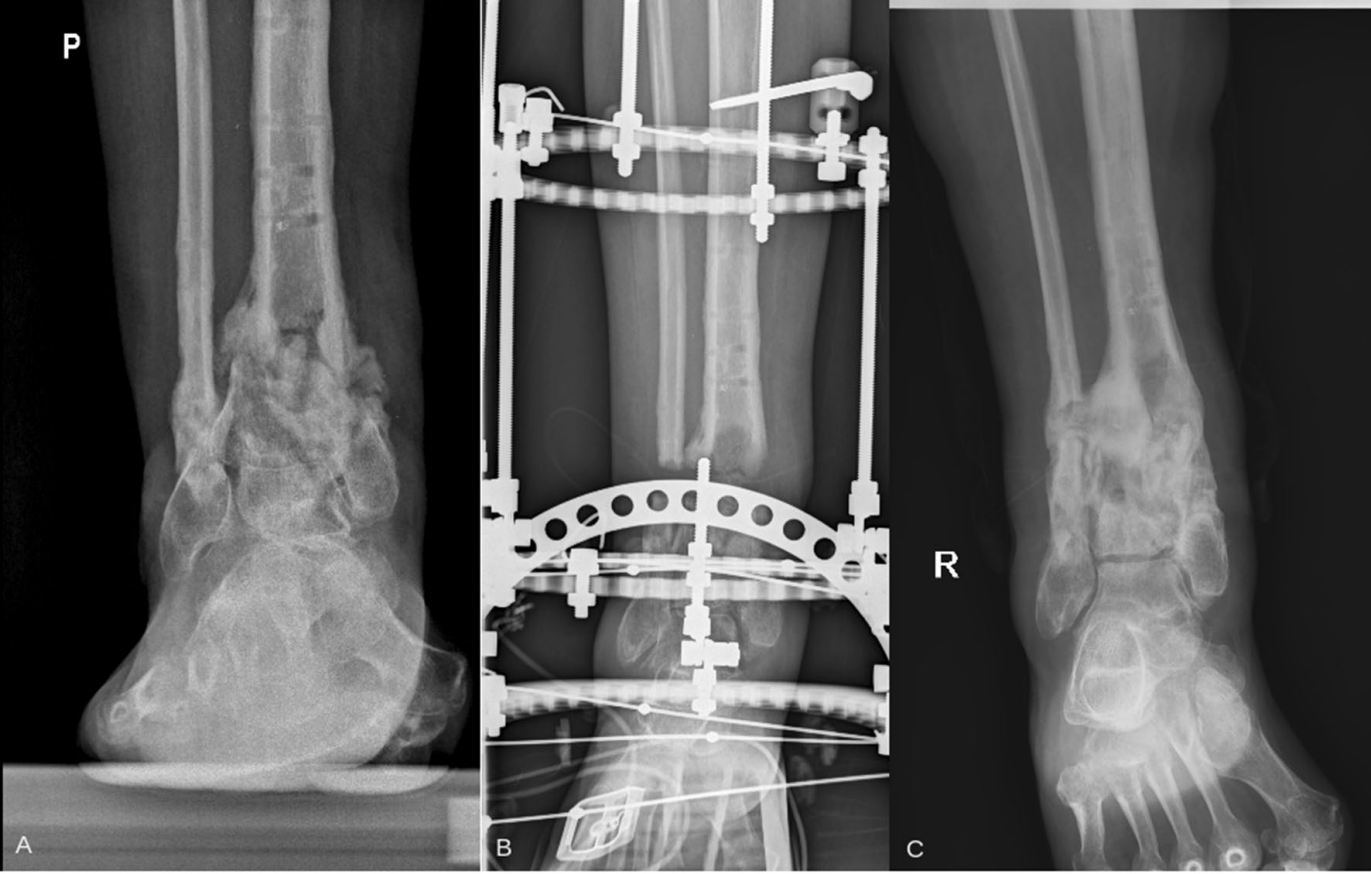
Patient with tibia nonunion. **a** X-ray from the pre-treatment period, **b** X-ray with Ilizarov apparatus after union, and **c** X-ray from the last follow-up visit after 7 years form apparatus removal, confirming maintained union

This study was a retrospective assessment of 102 patients treated by two of the authors for posttraumatic nonunion of the tibia in the period 2008–2015 (Figs. 1, 2).

The inclusion criteria were nonunion of the tibia treated with an Ilizarov external fixator, at least 2 years after treatment end, investigator access to all medical records, and radiological images relating to the patients’ treatment.

102 patients (26 women and 76 men) met all the inclusion criteria. Nonunion were caused failed previous internal plate fixation in 70 cases and failed previous intramedullary nail fixation in 32 cases (Table 1). The study was approved by the Local Institutional Review Board. It was single-center study. We analyzed medical and radiological documentation from hospital records.

**Table 1:** Patient characteristics

Patient number	Sex	Age	Type of nonunion	Location of nonunion	Causes of nonunion	Surgery technique
1	M	21	Hypertrophic	1/3 Mid	Failed previous plate fixation	Close
2	M	17	Hypertrophic	1/3 Mid	Failed previous intramedullary nail fixation	Close
3	M	48	Hypertrophic	1/3 Distal	Failed previous plate fixation	Close
4	M	41	Hypertrophic	1/3 Distal	Failed previous plate fixation	Close
5	M	73	Hypertrophic	1/3 Distal	Failed previous plate fixation	Open
6	M	29	Hypertrophic	1/3 Proximal	Failed previous plate fixation	Close
7	M	22	Hypertrophic	1/3 Distal	Failed previous plate fixation	Close
8	M	62	Atrophic	1/3 Distal	Failed previous plate fixation	Open
9	M	71	Hypertrophic	1/3 Mid	Failed previous intramedullary nail fixation	Open
10	M	38	Hypertrophic	1/3 Mid	Failed previous intramedullary nail fixation	Close
11	F	37	Hypertrophic	1/3 Distal	Failed previous plate fixation	Open
12	F	30	Hypertrophic	1/3 Mid	Failed previous plate fixation	Open
13	M	52	Hypertrophic	1/3 Mid	Failed previous intramedullary nail fixation	Open
14	M	53	Hypertrophic	1/3 Distal	Failed previous plate fixation	Close
15	M	30	Hypertrophic	1/3 Distal	Failed previous plate fixation	Close
16	M	42	Hypertrophic	1/3 Distal	Failed previous plate fixation	Close
17	M	60	Hypertrophic	1/3 Distal	Failed previous plate fixation	Close
18	M	54	Hypertrophic	1/3 Mid	Failed previous intramedullary nail fixation	Close
19	M	66	Hypertrophic	1/3 Proximal	Failed previous plate fixation	Close
20	M	50	Hypertrophic	1/3 Mid	Failed previous intramedullary nail fixation	Close
21	F	55	Hypertrophic	1/3 Distal	Failed previous plate fixation	Close
22	F	30	Hypertrophic	1/3 Proximal	Failed previous plate fixation	Close
23	M	51	Atrophic	1/3 Distal	Failed previous plate fixation	Close
24	M	50	Hypertrophic	1/3 Mid	Failed previous intramedullary nail fixation	Open
25	M	17	Hypertrophic	1/3 Distal	Failed previous plate fixation	Close
26	M	11	Hypertrophic	1/3 Mid	Failed previous plate fixation	Close
27	M	73	Hypertrophic	1/3 Mid	Failed previous intramedullary nail fixation	Close
28	F	71	Hypertrophic	1/3 Mid	Failed previous plate fixation	Close
29	F	23	Hypertrophic	1/3 Distal	Failed previous intramedullary nail fixation	Close
30	F	55	Atrophic	1/3 Proximal	Failed previous plate fixation	Open
31	F	60	Hypertrophic	1/3 Mid	Failed previous intramedullary nail fixation	Close
32	M	33	Hypertrophic	1/3 Distal	Failed previous plate fixation	Open
33	M	47	Hypertrophic	1/3 Mid	Failed previous intramedullary nail fixation	Open
34	M	33	Atrophic	1/3 Distal	Failed previous plate fixation	Close
35	F	23	Hypertrophic	1/3 Mid	Failed previous plate fixation	Close
36	M	61	Hypertrophic	1/3 Mid	Failed previous intramedullary nail fixation	Close
37	F	15	Hypertrophic	1/3 Distal	Failed previous plate fixation	Close
38	F	56	Hypertrophic	1/3 Distal	Failed previous plate fixation	Close
39	M	40	Atrophic	1/3 Distal	Failed previous plate fixation	Close
40	M	42	Atrophic	1/3 Distal	Failed previous plate fixation	Close
41	M	60	Hypertrophic	1/3 Mid	Failed previous intramedullary nail fixation	Open
42	M	34	Atrophic	1/3 Proximal	Failed previous plate fixation	Close
43	F	21	Atrophic	1/3 Mid	Failed previous intramedullary nail fixation	Open
44	M	48	Hypertrophic	1/3 Mid	Failed previous plate fixation	Close
45	M	41	Hypertrophic	1/3 Mid	Failed previous intramedullary nail fixation	Close
46	M	73	Hypertrophic	1/3 Distal	Failed previous plate fixation	Close
47	M	29	Hypertrophic	1/3 Distal	Failed previous plate fixation	Close
48	M	22	Hypertrophic	1/3 Distal	Failed previous plate fixation	Close
49	F	62	Atrophic	1/3 Proximal	Failed previous plate fixation	Open
50	F	71	Hypertrophic	1/3 Distal	Failed previous plate fixation	Close
51	M	77	Hypertrophic	1/3 Distal	Failed previous plate fixation	Close
52	M	38	Hypertrophic	1/3 Mid	Failed previous intramedullary nail fixation	Open
53	M	37	Hypertrophic	1/3 Mid	Failed previous intramedullary nail fixation	Open
54	M	30	Hypertrophic	1/3 Distal	Failed previous plate fixation	Close
55	F	52	Hypertrophic	1/3 Mid	Failed previous intramedullary nail fixation	Open
56	F	53	Hypertrophic	1/3 Mid	Failed previous intramedullary nail fixation	Close
57	M	30	Hypertrophic	1/3 Distal	Failed previous plate fixation	Close
58	M	42	Hypertrophic	1/3 Distal	Failed previous plate fixation	Close
59	M	56	Hypertrophic	1/3 Distal	Failed previous plate fixation	Close
60	M	54	Hypertrophic	1/3 Distal	Failed previous intramedullary nail fixation	Close
61	M	66	Hypertrophic	1/3 Mid	Failed previous plate fixation	Close
62	M	50	Hypertrophic	1/3 Proximal	Failed previous plate fixation	Close
63	M	55	Hypertrophic	1/3 Mid	Failed previous intramedullary nail fixation	Close
64	F	30	Atrophic	1/3 Distal	Failed previous plate fixation	Open
65	F	60	Hypertrophic	1/3 Proximal	Failed previous plate fixation	Close
66	M	54	Atrophic	1/3 Distal	Failed previous plate fixation	Close
67	M	51	Atrophic	1/3 Mid	Failed previous intramedullary nail fixation	Close
68	F	59	Hypertrophic	1/3 Distal	Failed previous plate fixation	Close
69	M	64	Hypertrophic	1/3 Mid	Failed previous intramedullary nail fixation	Close
70	M	54	Hypertrophic	1/3 Mid	Failed previous intramedullary nail fixation	Close
71	F	45	Hypertrophic	1/3 Mid	Failed previous plate fixation	Close
72	M	61	Hypertrophic	1/3 Distal	Failed previous plate fixation	Open
73	F	34	Atrophic	1/3 Proximal	Failed previous plate fixation	Close
74	M	48	Atrophic	1/3 Mid	Failed previous intramedullary nail fixation	Open
75	M	29	Hypertrophic	1/3 Distal	Failed previous plate fixation	Open
76	F	51	Hypertrophic	1/3 Proximal	Failed previous plate fixation	Close
77	M	53	Atrophic	1/3 Distal	Failed previous plate fixation	Close
78	M	15	Hypertrophic	1/3 Mid	Failed previous intramedullary nail fixation	Close
79	M	76	Hypertrophic	1/3 Mid	Failed previous intramedullary nail fixation	Close
80	M	64	Hypertrophic	1/3 Distal	Failed previous plate fixation	Close
81	M	53	Hypertrophic	1/3 Distal	Failed previous plate fixation	Close
82	M	77	Hypertrophic	1/3 Distal	Failed previous plate fixation	Close
83	M	25	Hypertrophic	1/3 Mid	Failed previous intramedullary nail fixation	Open
84	F	25	Atrophic	1/3 Distal	Failed previous plate fixation	Close
85	F	62	Hypertrophic	1/3 Proximal	Failed previous plate fixation	Open
86	M	54	Atrophic	1/3 Distal	Failed previous plate fixation	Close
87	M	48	Hypertrophic	1/3 Distal	Failed previous plate fixation	Close
88	M	45	Hypertrophic	1/3 Distal	Failed previous plate fixation	Close
89	M	53	Hypertrophic	1/3 Mid	Failed previous intramedullary nail fixation	Open
90	M	51	Hypertrophic	1/3 Distal	Failed previous plate fixation	Close
91	F	74	Hypertrophic	1/3 Distal	Failed previous plate fixation	Close
92	M	27	Atrophic	1/3 Mid	Failed previous intramedullary nail fixation	Open
93	M	77	Hypertrophic	1/3 Proximal	Failed previous plate fixation	Open
94	M	36	Hypertrophic	1/3 Distal	Failed previous plate fixation	Open
95	M	36	Hypertrophic	1/3 Mid	Failed previous intramedullary nail fixation	Open
96	M	29	Hypertrophic	1/3 Distal	Failed previous plate fixation	Close
97	M	34	Atrophic	1/3 Distal	Failed previous plate fixation	Close
98	M	38	Hypertrophic	1/3 Distal	Failed previous plate fixation	Close
99	M	48	Hypertrophic	1/3 Distal	Failed previous plate fixation	Open
100	M	38	Hypertrophic	1/3 Distal	Failed previous plate fixation	Close
101	F	65	Atrophic	1/3 Distal	Failed previous plate fixation	Close
102	M	59	Hypertrophic	1/3 Mid	Failed previous intramedullary nail fixation	Open

Patient number	ASAMI bone score	ASAMI functional score	Refracture	Follow-up (years)
1	Excellent	Excellent	No	5.6
2	Excellent	Excellent	No	11.7
3	Excellent	Excellent	No	11.3
4	Good	Good	No	11.2
5	Excellent	Excellent	No	11
6	Excellent	Good	No	10.9
7	Excellent	Excellent	No	10.5
8	Excellent	Good	No	10.4
9	Excellent	Excellent	No	10.1
10	Excellent	Excellent	No	10
11	Excellent	Good	No	9.6
12	Excellent	Good	No	9.7
13	Excellent	Excellent	No	9.4
14	Excellent	Excellent	No	9.6
15	Excellent	Excellent	No	9.5
16	Excellent	Excellent	No	9.2
17	Excellent	Good	No	9
18	Excellent	Good	No	8.2
19	Excellent	Excellent	No	7.7
20	Good	Good	No	8.5
21	Excellent	Excellent	No	8.4
22	Excellent	Excellent	No	10.9
23	Excellent	Excellent	No	8.5
24	Excellent	Good	No	11.7
25	Excellent	Excellent	No	11.2
26	Excellent	Excellent	No	11.5
27	Excellent	Excellent	No	11.7
28	Excellent	Excellent	No	12
29	Excellent	Good	No	11.7
30	Excellent	Excellent	No	11.5
31	Excellent	Good	No	11.7
32	Excellent	Excellent	No	2.5
33	Excellent	Excellent	No	10.6
34	Excellent	Good	No	11.6
35	Excellent	Good	No	12
36	Excellent	Excellent	No	11.9
37	Excellent	Excellent	No	10.8
38	Good	Good	No	11.8
39	Excellent	Excellent	No	10.5
40	Excellent	Excellent	No	11.6
41	Excellent	Excellent	No	11.5
42	Good	Good	No	10.8
43	Excellent	Excellent	No	11.8
44	Excellent	Excellent	No	7.41
45	Good	Good	No	5.3
46	Excellent	Excellent	No	2.01
47	Excellent	Good	No	8.98
48	Excellent	Excellent	No	8.54
49	Excellent	Good	No	10.01
50	Excellent	Excellent	No	7.03
51	Excellent	Excellent	No	5.96
52	Excellent	Good	No	5.87
53	Excellent	Good	No	3.99
54	Excellent	Excellent	No	4.72
55	Excellent	Excellent	No	3.44
56	Excellent	Excellent	No	3.68
57	Excellent	Excellent	No	3.5
58	Excellent	Good	No	2.39
59	Excellent	Good	No	5.86
60	Excellent	Excellent	No	4.28
61	Good	Good	No	3.13
62	Excellent	Excellent	No	2.56
63	Excellent	Excellent	No	8.53
64	Excellent	Excellent	No	2.06
65	Excellent	Excellent	No	2.31
66	Good	Good	No	5.81
67	Excellent	Excellent	No	2.4
68	Excellent	Good	No	2.21
69	Poor	Poor	Yes	2.85
70	Excellent	Excellent	No	2.25
71	Excellent	Excellent	No	2.07
72	Excellent	Good	No	2.51
73	Excellent	Excellent	No	2.16
74	Excellent	Excellent	No	2.3
75	Excellent	Excellent	No	4.4
76	Excellent	Excellent	No	6.33
77	Poor	Poor	Yes	10.5
78	Excellent	Excellent	No	7.5
79	Excellent	Excellent	No	8.5
80	Excellent	Excellent	No	4.5
81	Excellent	Good	No	7.66
82	Excellent	Excellent	No	8
83	Excellent	Excellent	No	6.66
84	Poor	Poor	Yes	6.5
85	Excellent	Excellent	No	6.5
86	Excellent	Good	No	6.58
87	Excellent	Excellent	No	5.5
88	Excellent	Excellent	No	4.5
89	Excellent	Excellent	No	4.58
90	Excellent	Excellent	No	2.58
91	Excellent	Good	No	3.33
92	Excellent	Excellent	No	2.5
93	Good	Good	No	2.58
94	Excellent	Excellent	No	2.5
95	Excellent	Excellent	No	2.66
96	Excellent	Excellent	No	6.42
97	Poor	Poor	Yes	2.75
98	Excellent	Excellent	No	2.5
99	Excellent	Excellent	No	2.5
100	Excellent	Excellent	No	2.66
101	Poor	Poor	Yes	2.58
102	Excellent	Excellent	No	2.58

The surgical procedures were conducted by two experienced orthopedic surgeons. In the case of nonunion located in the proximal two-thirds of the tibial shaft, the Ilizarov apparatus consisted of four rings fixed to the tibia and fibula with Kirschner wires. In the case of nonunion of the distal third of the tibia, the Ilizarov apparatus consisted of three rings fixed to the tibia and fibula with Kirschner wires and a foot frame stabilized with three olive Kirschner wires. Tibial nonunion were treated with stabilization and compression, without the use of the bone transport technique. In 73 cases, closed stabilization of nonunion was performed. In 29 patients an open, small bone fragment resection was performed, with adaptation of the nonunion edges and stabilization with the Ilizarov apparatus. We did not use bone grafts. The distal surface of the proximal tibial fragment and the proximal surface of the distal tibial fragment were drilled with Kirschner wires according to Becks’s method.

Patient verticalization and gait training with partial weight-bearing on the operated limb and the use of two forearm crutches was initiated on postoperative day one. Clinical and radiographic follow-up visits were conducted in an outpatient setting in 2–6-week intervals. Over the course of treatment, loading of the operated limb was progressive increased until, eventually, the crutches could be discarded as full weight-bearing was achieved.

The Ilizarov external fixator was removed once union of the nonunion was confirmed radiographically and clinically. The radiographic criterion of union was the presence of at least three out of four cortices or trabecular bridging in anteroposterior and lateral views. The clinical criteria were the absence of pain, absence of pathological mobility, and absence of lower leg deformity on dynamization of the Ilizarov apparatus or on forcible attempts at movement at the site of nonunion. Once their Ilizarov external fixator was removed, the patients were advised to walk with two forearm crutches and bear partial weight on the operated limb for 4 weeks. Loading of the limb was gradually increased, depending on the degree of bone remodeling at the site of nonunion visualized with radiography.

Study assessments were based on radiographic images obtained during treatment and at a follow-up visit minimum 2 years after the removal of Ilizarov external fixator. The assessed parameters were bone union achieved during treatment, duration of stabilization with an Ilizarov external fixator, and maintained bone union at the last follow-up visit. The Association for the Study and Application of the Method of Ilizarov (ASAMI) bone score and ASAMI functional score were evaluated as well at the last follow-up visit [28, 29].

The statistical analysis was conducted with STATISTICA 13.3 software. The Shapiro–Wilk test was used to evaluate the normality of distribution of all quantitative parameters. The Mann–Whitney *U* test or Kruskal–Wallis test (ANOVA) was used to calculate differences between groups. The Wilcoxon signed-rank test for paired samples was used for repeated measurements; potential correlation was assessed with Spearman’s rank correlation coefficient (rho). The level of statistical significance was set at *p* < 0.05.

## Results

A total of 102 patients were assessed (Table 1). The mean age at the start of treatment was 46.5 years (11–77 years, SD 17.35). The mean follow-up period was 7 years (2–12 years, SD 2.23). Bone union was achieved in all patients. The mean duration of Ilizarov stabilization in the study group was 7.9 months (2.8–20.7 months, SD 4.29). ASAMI bone scores were excellent in 88 cases, good in nine cases, and poor in five case. ASAMI functional scores were excellent in 67 cases, good in 30 cases, and poor in five case. The rate of union maintained at the last follow-up visit was 95.1% (i.e., bone union was maintained in 97 out of 102 evaluated patients). Five persons developed a re-fracture of the healed site and required restabilization with an Ilizarov external fixator. Four out of five patients with re-fracture had infected, atrophic nonunion in 1/3 distal of tibia. All of patients with re-fracture had close surgery technique. The results of statistical analysis showed that infection (*p* = 0.032), atrophic nonunion (*p* = 0.021), nonunion in 1/3 distal of tibia (*p* = 0.038), and close surgery technique (*p* = 0.017) are the independent risk factors of re-fracture. In the hypertrophic pseudarthrosis group, the median time to union (195.0 days) was significantly shorter than in the atrophic pseudarthrosis group (299.0 days), *p* = 0.021. The mean time of re-fracture was 2 months after Ilizarov fixator removal (1–6 months). The mean time to union after Ilizarov re-fixation was 9.4 months (4.8–12.7 months, SD 3.34). All of the re-fracture patients had poor results in ASAMI Bone Score and ASAMI Functional Score.

At least one risk factor for disturbance in bone healing has been reported in 22 patients. The following risk factors were considered: corticosteroid therapy, smoking, alcohol dependence, diabetes mellitus, and advanced lower-extremity vascular disease. There was no significant difference in time to union between the group of patients with risk factor for disturbance in fracture healing and the group without risk factors (*p* = 0.827).

## Discussion

Nonunion of the tibia is a common treatment complication in tibial fractures [3–6]. Despite advancements in surgical techniques, nonunion remains a serious therapeutic issue [2–7, 9, 10, 12–19], as it often requires comprehensive surgical treatment involving resection of damaged bone and soft tissues, excision of the focus of infection, and secondary elongation and realignment of the affected bone segment following bone transport [1–7, 9–19]. While evaluating treatment outcomes in nonunion of the tibia, the majority of authors focus on whether or not bone union was achieved [1–27, 30–33]. However, bone union may be only short term, as some patients develop re-fracture [1, 19], which necessitate further treatment and adversely affect long-term outcomes.

The purpose of our study was to assess the rates of achieved (short term, achieving union after treatment) and maintained (long term) bone union and determination of re-fracture factors in a group of patients treated at the Our Clinic, to review the literature on the treatment of nonunion of the tibia with Ilizarov external fixators, and to compare the results reported in the available literature with our findings. We include a larger cohort of tibial nonunion cases (102), whether aseptic or infected, to study the incidence of re-fracture after long-term follow-up of Ilizarov fixation and to address different risk factors, treatment methods, and the effect of re-fracture on final outcome in comparison to cases with maintained bone union.

Yin et al. conducted a meta-analysis of 24 studies in a total of 590 patients treated with an Ilizarov external fixator due to infected femoral or lower leg nonunion [1] and showed a union rate of 97.8%. Six of the analyzed studies considered re-fracture as one of treatment complications and reported it in a mean of 4% of cases [1]. However, the authors did not include any information on when the re-fracture occurred, its treatment, or its effect on the treatment outcome. The proportion of patients in whom bone union was successfully maintained was likewise not included [1]. Peng et al. presented 58 cases of treating infected nonunion of the tibia with Ilizarov bone transport with an antibiotic-loaded bone cement spacer. Bone union was achieved in all patients [2]. Ilizarov fixator were removed after a 10.6 months. They have no re-fracture in follow-up. McNally et al. evaluated 79 patients with nonunion of the tibia treated with the Ilizarov method implemented with various surgical techniques [19]. Depending on the surgical technique used, primary bone union was achieved in 73.7–96.2% of patients, with the re-fracture rate in the monofocal compression group as high as 31.6% [19]. Further treatment helped to achieve union in 100% of cases. The authors did not assess the proportion of patients who achieved maintained bone union [19]. Laursen assessed 16 patients with nonunion of the tibia treated with the Ilizarov method and reported bone union in 93.8% of patients after a mean treatment duration of 6 months, with no recorded cases of re-fracture [25].

In the literature reports about tibial nonunion treatment, only a few authors give short information about protocol in preventing re-fracture [5, 7, 12, 13, 17, 21]. Abuomira et al. evaluated 55 patients treated with circular frames due to nonunion of the tibia [7]. Bone union was achieved in 89% of cases, with the mean treatment duration of 13 months. They removed external fixator when three or four cortical was seen in radiographs. After external fixator removal, patients walked with partial weight-bearing for 4–6 weeks. The rates of maintained union were not reported [7]. Madhusudhan et al. evaluated 22 patients treated with Ilizarov external fixators due to nonunion of the tibia. Bone union was achieved in 81.8% of patients [12]. They removed external fixator when union was seen in radiographs. After external fixator removal patients walked with functional cast brace for a few weeks [12]. Magadum, who analyzed treatment outcomes in 25 patients with nonunion of the tibia treated with an Ilizarov external fixator, reported bone union in 96% of patients [13]. After Ilizarov fixator removal patients walked with cast for a 6 weeks [13]. Meleppuram et al. achieved bone union in 100% out of 42 patients with nonunion of the tibia treated with an Ilizarov external fixator [5]. They removed fixator when the nonunion was corticolized on 3 of 4 sides. They used casts for a 2 months [5]. Wang et al. assessed 15 patients with nonunion of the tibia treated with circular frames. After a mean of 12 months, bone union was achieved in 100% of cases [17]. They dynamized the frame before removal for assess the mechanical stability of the new bone. They removed fixator when the nonunion was corticolized on 3 of 4 sides in radiographs. After external fixator removal, they applied functional brace for at least 4 weeks [17]. Yin achieved union in all of the 60 patients with nonunion of the tibia treated with an Ilizarov external fixator [21]. Yin removed external fixator when radiographs showed a minimum of three complete cortices [21].

We believe that very good outcome in our patients (re-fracture only in 4.9% of patients) are related to our treatment protocol. We have minimized the risk of re-fracture through delayed frame removal and weight-bearing protocol. The average time of Ilizarov frame removal was 7.9 months. Ilizarov external fixator was removed once union of the nonunion was confirmed radiographically and clinically. The weight-bearing protocol is also important. Loading of the limb was gradually increased, depending on the degree of bone remodeling at the site of nonunion visualized with radiography.

The authors presented papers in which they evaluated from 8 to 94 patients treated with circular frames due to nonunion of the tibia [2–27, 30, 32]. In our work, we evaluated a group of 102 patients. All patients in our study achieved bone union, which constitutes a better outcome than those reported on average in the literature (73.7–100%) [1–27, 30, 32]. Callus formation and bone union tend to take a longer time in patients with nonunion of the tibia [10, 13]. This extends treatment duration (the length of time which the Ilizarov apparatus remains on the treated limb) in comparison to that in patients who undergo corrective surgeries, such as limb lengthening [10, 13]. This is another reason why patients with nonunion of the tibia treated with the Ilizarov method should be followed up for a longer time and why long-term treatment outcomes should be assessed. The mean length of time which the Ilizarov external fixator was in place in our patients was 7.9 months, which is consistent with the data from the literature (as the reported treatment duration ranged from 5.8 to 13.5 months) [3, 4, 7–11, 14, 17, 25].

None of the studies mentioned above [1–27, 30–33] assessed the proportion of patients with maintained bone union. In our study, maintained bone union was observed in 95.1% of patients at the follow-up visit at least 2 years after treatment, which indicates excellent long-term treatment outcomes in nonunion of the tibia treated with the Ilizarov method.

Four out of five patients with re-fracture had infected, atrophic nonunion in 1/3 distal of tibia. All of patients with re-fracture had close surgery technique. Infection, atrophic nonunion, nonunion in 1/3 distal of tibia, and close surgery technique are a risk factors of re-fracture after tibia nonunion treatment with Ilizarov method. All of the re-fracture patients had poor results in ASAMI Bone Score and ASAMI Functional Score. Re-fracture has a negative effect on the final outcome in comparison to cases with maintained bone union.

## Conclusions

Since long-term treatment outcomes are the most importance to both the patients and the surgeons, we suggest that maintained union rates be introduced as a new assessment criterion of long-term outcomes. It is both the union rates (short term, achieving union after treatment) and maintained union rates (long term) that need to be assessed as part of any comprehensive evaluation of Ilizarov treatment outcomes in patients with nonunion of the tibia.

The Ilizarov method helps to achieve very good short-term and long-term outcomes both in the treatment of nonunion of the tibia.

Infection, atrophic nonunion, nonunion in 1/3 distal of tibia, and close surgery technique are a risk factors of re-fracture after tibia nonunion treatment with Ilizarov method.
